# Evaluation of the efficacy of CT-guided 3D template-assisted ^125^I seed implantation in the treatment of unresectable STS: a multicenter retrospective study

**DOI:** 10.1038/s41598-022-07729-9

**Published:** 2022-03-08

**Authors:** Guang Sheng Zhao, Song Liu, Hua Liu, Wen Cai Lv, Liang Yang, Chuang Li, Jun Zhou, Ruo Yu Wang, Yong Chun Song

**Affiliations:** 1 Cancer Treatment Center, Affiliated Zhongshan Hospital of Dalian University, No.6 Jie Fang Street, Dalian, 116001 Liaoning Province China; 2 Interventional Medicine Center, Linyi Cancer Hospital, 6 East Lingyuan Street, Linyi, 276001 Shandong Province China; 3 Department of Surgical Oncology, First Affiliated Hospital of Xi’an Jiaotong University, 277 West Yanta Road, Xi’an, 710061 Shaanxi Province China; 4 Dalian Friendship Hospital of Dalian Medical University, No. 8, Sanba Square, Zhongshan District, Dalian, 116100 Liaoning Province China

**Keywords:** Cancer, Diseases, Medical research, Oncology

## Abstract

To observe the safety and efficacy of CT-guided 3D template-assisted radioactive ^125^I seed implantation in the treatment of unresectable advanced soft tissue sarcoma (STS). Sixty-two patients who underwent continuous 3D template-assisted radioactive ^125^I seed implantation for the treatment of unresectable advanced STS from August 2017 to August 2018 were selected from four tumor treatment centers for retrospective analysis. The postoperative adverse reactions and tumor response were recorded, and the postoperative complications were observed and treated at the same time. The overall survival (OS) rate was determined. All patients successfully completed ^125^I seed implantation. In practice, the median number of puncture needles used during the operation was 20, and the median number of ^125^I particles was 88. There were no statistical differences in the relative dosimetry parameters before and after the operation (P > 0.05). Tumor evaluation was performed 6 months after the operation. The effective rate was 61.3%, and the local control rate was 93.5%. As of March 2020, the 1-year survival rate was 85.2%, and the 2-year survival rate was 49.0%. The OS was 23 months. CT-guided 3D template-assisted ^125^I seed implantation for the treatment of unresectable STS has a high local control rate, thereby further prolonging the OS of patients with unresectable STS.

## Introduction

Soft tissue sarcoma (STS) is a type of pluripotent stem cell cancer originating in the mesenchymal tissue of the mesoderm^1,2^, and it includes nerve tissue tumors in the neuroectoderm but excludes those in bone, cartilage, and lymphoid hematopoietic tissues. Its incidence is approximately 1.28/100,000–1.72/100,000, which accounts for only 0.73–0.81% of all malignant tumors in adults. However, the prognosis is poor, especially for patients with unresectable advanced STS and STS recurrence after surgery. The overall survival (OS) is approximately 6–18 months^3,4^. STS can occur in individuals of any age, with a slightly higher prevalence in males than females, and 50–60% of all cases occur in the limbs and 5% occur in the head and neck. Undifferentiated pleomorphic sarcoma is the most common type of STS (25–35%), and it is common in middle-aged and elderly individuals, where it has a high rate of regional lymph node metastasis. Embryonic rhabdomyosarcoma grows the fastest, followed by undifferentiated pleomorphic sarcoma. The better-differentiated mucoliposarcoma grows slowly, and the prognosis of head and neck STS is poor. In patients with high-grade STS, metastasis has already occurred in 10% of cases at the time of initial diagnosis. Even if the tumor is locally controlled, 40–50% of patients will have local recurrence after the surgery, and > 50% will have distant metastasis. The most common site of distant metastasis is the lung (50%)^1,2,8^.

Surgery is the only cure for most patients with STS. The purpose of surgery is not only to completely remove the tumor, but also to obtain a safe surgical margin^1,4,8,9^. When R0 resection cannot be achieved, preoperative radiotherapy combined with chemotherapy and interventional therapy are required; otherwise, amputation is needed at the expense of partial function. Local extensive resection combined with adjuvant radiotherapy is the standard treatment mode for the surgical resection of high-grade STS, and the curative effect of radiotherapy depends on the pathological type of STS and tumor burden^10–12^. Studies have shown that the surgical margin after the surgery greatly affects the prognosis and quality of life^13^. Mo et al. reported that the OS of ^125^I seed implantation combined with second-line chemotherapy for the treatment of metastatic STS was 16.9 months, and the 6-month local control rate was 62.2%^5^. With the clinical application of 3D printing technology, 3D-template assisted ^125^I seed implantation greatly improves the treatment accuracy of malignant tumors, especially advanced refractory malignant tumors^6,7^. However, there is no study on the application of 3D-template assisted ^125^I seed implantation in the treatment of advanced unresectable STS. The purpose of this study is to further explore the clinical application value of this technology in this field.

## Clinical data and methods

### General information

From August 2017 to August 2018, 62 patients (34 males, 28 females) with STS were selected from four tumor treatment centers, namely, Dalian University affiliated Zhongshan Hospital, First Affiliated Hospital of Xi'an Jiaotong University, Beijing Tsinghua Changgung Hospital, and Linyi Cancer Hospital. The patients’ ages ranged from 38 to 84 years (median age, 55 years; average age, 54.8 ± 14.2 years). The inclusion criteria were as follows: (i) patients with postoperative recurrence or unresectable STS, (ii) patients with tumor progression after radiotherapy or chemotherapy, and (iii) patients with advanced STS without radiotherapy or chemotherapy. All patients voluntarily accepted the treatment method and signed a consent form for seed implantation surgery. This study was approved by the ethics committee of our hospital. The clinical data of the patients are given in Table 1.

**Table 1:** Clinical data of 62 cases of STS.

Item	Number	Proportion
**Gender**		
Male	34	54.8
Female	28	45.2
**Pathological type**		
Leiomyosarcoma	14	22.6
Neurogenic sarcoma	11	17.7
Malignant fibrous histiocytoma	9	14.5
Rhabdomyosarcoma	4	6.5
Synovial sarcoma	3	4.8
Epithelioid sarcoma	2	3.2
Fibrosarcoma	7	11.3
Liposarcoma	9	14.5
Others	5	8.1
**Pathological grade**		
G1	27	43.5
G2	18	29.0
G3	17	27.4
**Tumor location**		
Head and neck	20	32.3
Body	13	21.0
Abdominal cavity retroperitoneum	11	17.7
Limbs	18	29.0
**Previous surgery**		
R0	40	64.5
R1	15	24.2
R2	3	4.8
None	4	6.5
**Previous radiotherapy**		
Yes	43	69.4
No	19	30.6
**Previous chemotherapy, targeted therapy**		
Yes	39	62.9
No	23	37.1
**Complicated with multiple metastases or not**		
Yes	24	38.7
No	38	61.3

### Materials and equipment

Domestic radioactive ^125^I seeds having a half-life of 60.2 d, an activity of 0.6–0.8 mCi (1ci = 3.7 × l010 Bq), and a γ-ray energy of 27–35 keV were used. The brachytherapy treatment planning system (BTPS) (Beijing Astro Technology Ltd., Co., Beijing, China) was used, combined with puncture needles (Hakko Co., Ltd., Japan), the TRH-BXQ implant gun (China), the TRH-J implant positioning device, and the GE 16-row spiral CT instrument.

### Preparation of preoperative plan with BTPS

Parameters, such as planned target dose (PTD), particle activity, and CT data, were input into the BTPS to simulate the needle insertion, develop the preoperative plan, and derive various parameters. The preoperative plan was conceived by the operator and the physicist after a thorough discussion, and each patient’s imaging position was adjusted to the actual operating position to ensure that the intra-operative needle insertion was consistent with the preoperative plan. The planned target area prescription dose (PD) was controlled within a range of 110–180 Gy.

### ^125^I seed implantation technology

Local and intravenous anesthesia was administered, and the positioning navigation device was installed according to the surface marking laser positioning line and adjusted to the template. The positions and angles of the inserted needles were controlled, and needle channels were established according to the preoperative plan. Starting from the center plane of the tumor, the needles were arranged in layers with a lateral margin of 1 cm and a depth of 0.5 cm from the distal edge. The particles were more than 1 cm away from the skin to avoid damage to the skin. If necessary, intraoperative planning correction and target dose optimization were performed.

### Postoperative dose verification

Additional parameters were input into the BTPS for particle reconstruction and postoperative dose verification. The relevant dosimetric parameter of the planned target volume (PTV) 1 cm outside of the clinical target volume (CTV) before and after surgery was calculated, including the doses of 90% of the target volume (D90), 90% of the prescription dose (PD) covered target volume (V90), 100% PD covered target volume (V100), 150% PD covered target volume (V150), the conformal index (CI), and the external index (EI) of the target. The dose parameters before and after the surgery are given in Table 2.

**Table 2:** Comparison of the consistency of metrological parameters in the target area before and after surgery in 62 cases of STS (median value).

Plan	Number of needles	Particle number	D90 (Gy)	D100 (Gy)	V100 (Gy)	
Preoperative	24 (6–70)	76 (23–201)	155 (93–207)	71 (55–95)		92 (91–94)
Postoperative	20 (6–62)	88 (22–206)	156 (95–201)	70 (52–96)		91 (90–93)
Z value	0.785	0.069	0.147	0.506		0.763
P value	0.442	0.967	0.902	0.625		0.474
Plan	V150 (%)	V200 (%)	CI	EI		HI
Preoperative	58.5 (51.0–61.5)	25.0 (18.9–30.8)	0.728(0.600–0.790)	0.208 (0.150–0.290)		0.348 (0.302–0.401)
Postoperative	55.3 (47.2–60.8)	22.8 (17.5–28.9)	0.720(0.598–0.775)	0.202 (0.146–0.288)		0.369 (0.345–0.421)
Z value	0.600	1.067	0.756	0.302		0.537
P value	0.567	0.311	0.498	0.781		0.597

### Postoperative treatment and observation

The postoperative Karnofsky physical score (KPS) and related adverse reactions were recorded. Adverse reactions were evaluated according to the Radiation Therapy Oncology Group/European Research Organization for the Treatment of Cancer (RTOG/EROTC) radiation damage grading criteria; pain was scored according to the Numerical Score (NAS). The tumor response was followed-up for 6 months according to the Response Evaluation Criteria of Solid Tumors (version 1.1). The survival time was observed and summarized, and the follow-up was concluded in March 2019 or at the time of patient death.

### Statistical analysis

Statistical analysis was performed using SPSS 20.0 software (IBM, Armonk, NY, USA). Data were expressed as mean ± standard deviation ($\overline{x} \pm s$). Student’s *t*-test was used for comparisons of normally distributed data between groups. The rank sum test was used for comparisons of non-normally distributed data between groups. The r test was used for comparisons of count data between groups. The Kaplan–Meier method was used to determine the survival rate. P < 0.05 was considered statistically significant.

### Ethics approval and consent to participate

The study was approved by the Ethics Committee of Affiliated Zhongshan Hospital of Dalian University.

### Consent for publication

All the patients participate in the study have signed the informed consent.

### Statement

All authors confirm that all methods were carried out in accordance with relevant guidelines and regulations.

## Results

### Comparison of pre- and post-operative dosimetric parameters

**Figure 1:**
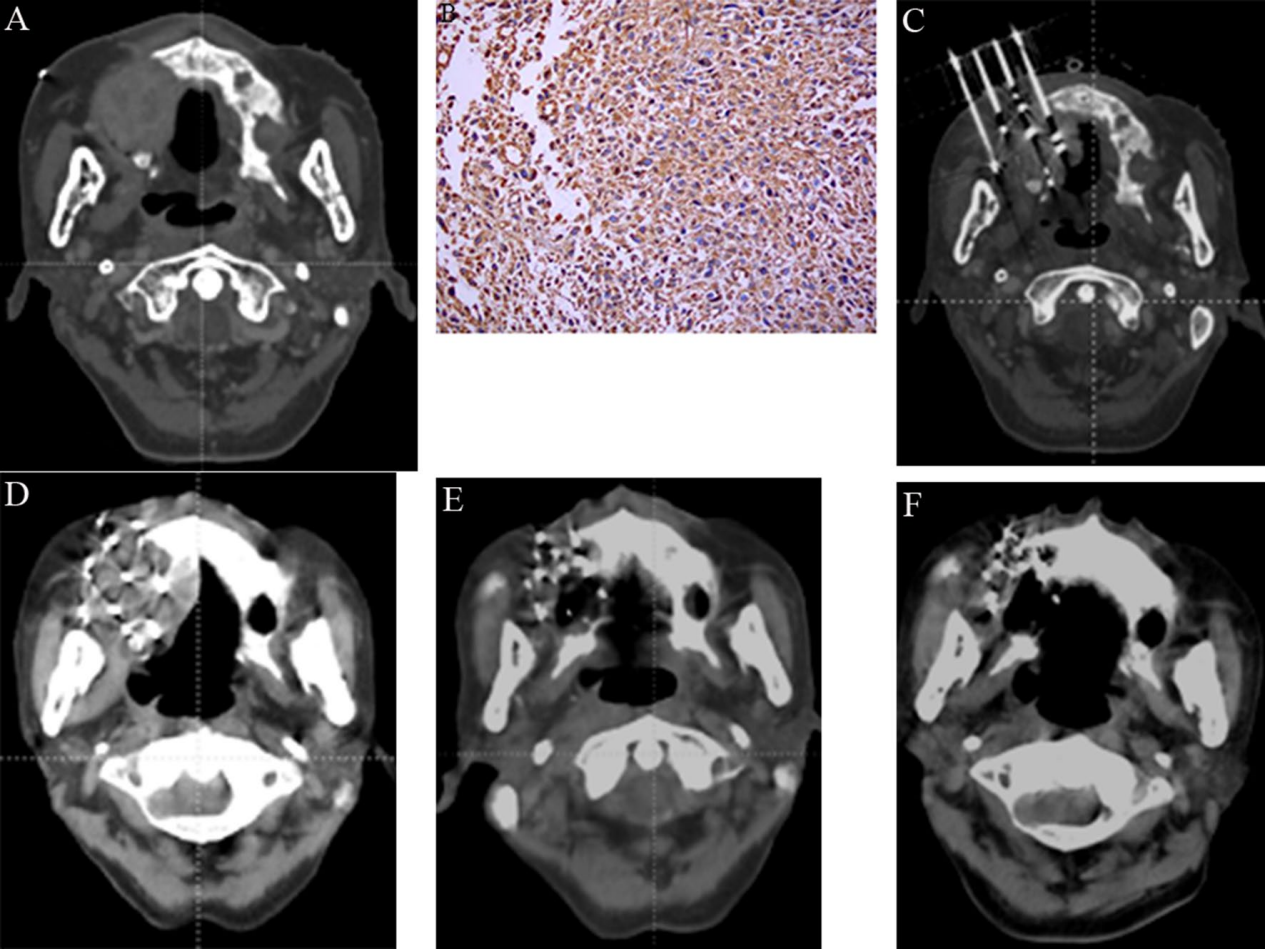
(**A**) Preoperative CT image of an 81-year-old male patient with maxillary sarcoma showed the tumor invaded the surrounding tissues. (**B**) Preoperative biopsy pathological pictures suggesting the undifferentiated sarcoma (or malignant fibrous histiocytoma). (**C**) In interventional surgery, the template-assisted CT-guided needle insertion image showed that the needle insertion angle and depth were good, and the large blood vessels were successfully avoided. (**D**): Re-examination at 3 days after intervention, CT showed uniform particle distribution in the lesion area. (**E**) CT image at 6 months after seed implantation showed that the lesion was significantly reduced, and a small amount of residual lesions could be observed on the edge. (**F**) At 1 year after intervention, CT image showed that the lesion disappeared completely, the boundary was clear, and the implanted particles gathered locally.

Under the guidance of 3D template-assisted CT, seed implantation was successfully completed in all patients. Dosage optimization was performed during the operation, and dose verification was performed after the operation. The median number of implanted puncture needles after the operation was 20, and the median number of implanted seeds was 88. There were no significant differences in the other related dosimetric parameters (P > 0.05), and the postoperative verification results were considered satisfactory, as shown in Table 2. Figure 1 shows the surgical ^125^I seed implantation procedure in one case of senile fibrous histiocytoma and the follow-up imaging.

### Complications and tumor response after seed implantation

After the operation, two patients with elbow sarcoma developed skin ulcerations and received symptomatic treatment. The scars healed gradually in approximately 6 months. There were no serious complications such as uncontrollable bleeding and puncture implant metastasis. CT or MRI was conducted 1, 2, 4, and 6 months after the surgery for dynamic imaging observation. The tumor response was systematically evaluated 6 months after the surgery, together with the characteristics of radioactive ^125^I seed attenuation. The tumor responses 6 months after the surgery were as follows: complete remission (CR), 19.4% (15/62); partial remission (PR), 53.2% (33/62); stable disease (SD), 20.9% (13/62); progressive disease, 6.5% (4/62); the objective remission rate (ORR), 61.3% (38/62); and the disease control rate (DCR), 93.5% (58/62) (Table 3). The adverse reactions of the cases we observed were mild and controllable after seed implantation treatment, and 2 cases of severe reactions were skin ulcers (1 case of 2nd degree, and 1 case of 3rd degree), which were relieved by symptomatic treatment without affecting the survival of the patients.

**Table 3:** Evaluation of tumor response in patients 6 months after seed implantation.

CR		PR		SD		PD		ORR		DCR	
n	%	n	%	n	%	n	%	n	%	n	%
15	19.4	33	53.2	13	20.9	4	6.5	38	61.3	58	93.5

### Adverse reactions

About 9.7% (6/62) of the patients had grade 1–2 skin adverse reactions, and no special treatment was given, and the patients improved spontaneously within about 2 weeks. Grade 3–4 skin adverse reactions (skin ulcers) occurred in 4.8% (3/62) of patients. Among them, 2 cases were considered to be the reaction to particle radiotherapy, and symptomatic treatment was actively given, and the scars were gradually healed in about 6 months; the other 1 case was considered to be caused by tumor progression and was not included in the treatment-related adverse reactions. Two patients (3.2%) experienced pain after seed implantation, and 1 patient (1.6%) developed transient fever.

### Statistics on the survival time

**Figure 2:**
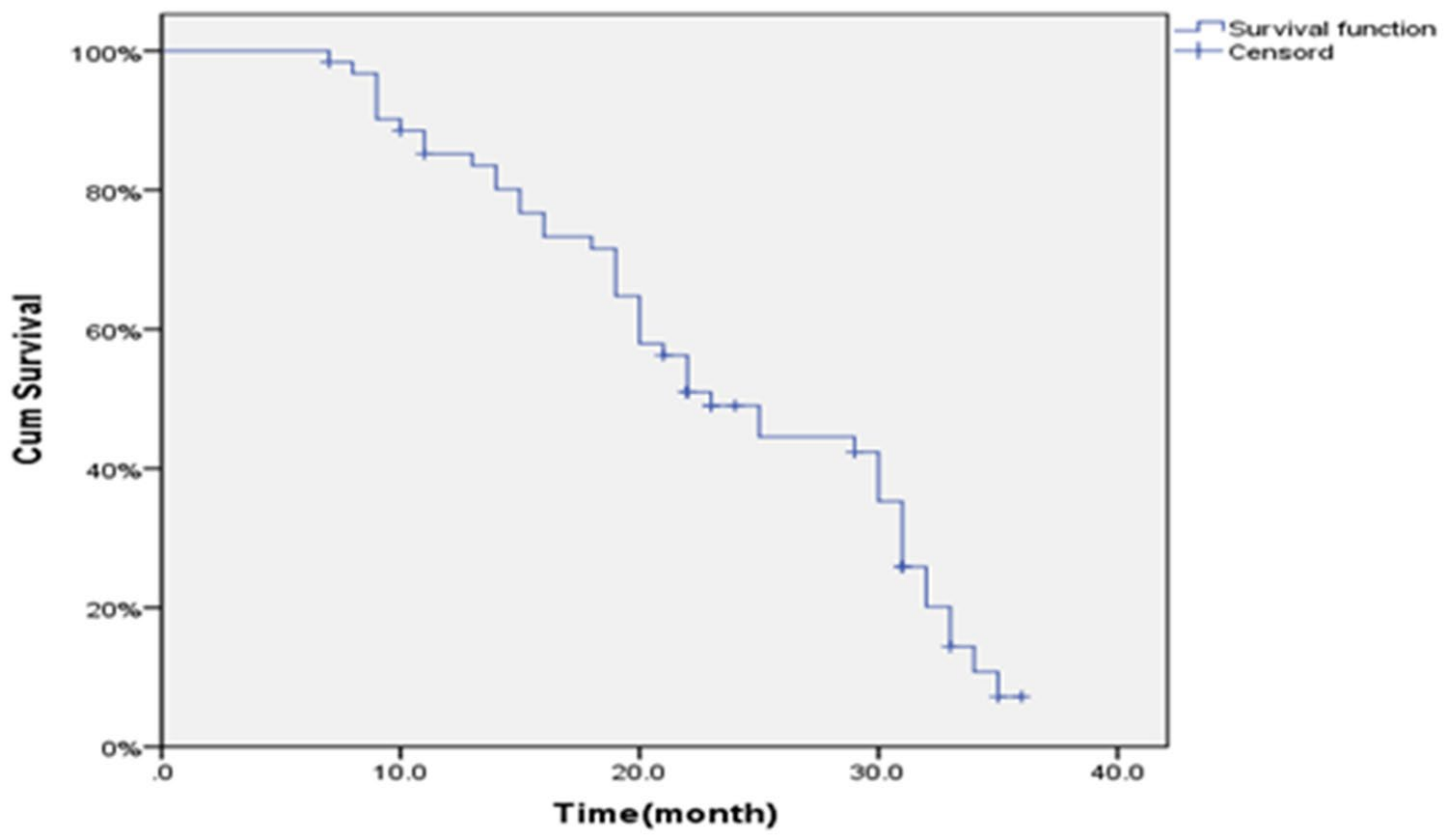
Survival curve of 62 cases of STS after seed implantation.

No patient in either group was lost to follow-up. All patients were followed-up according to the plan. As of March 2020, the follow-up time was 7–36 months (21.64 ± 8.55), and the OS was 23 months (95% Cl 18.598–27.402) (Fig. 2). The 1-year survival rate was 85.2%, and the 2-year survival rate was 49.0%.

## Discussion

Palliative radiotherapy combined with sequential chemotherapy, intervention, or other methods can be used for the treatment of locally advanced unresectable STS to prolong the OS^12–17^. A few patients with sensitive STS have a high response rate to chemotherapy, while the chemotherapy tolerance of patients with advanced senile STS is significantly reduced. Molecular targeted therapy has no indications of adjuvant and neoadjuvant therapy for STS, and it is mainly used as the second- or third-line treatment of advanced unresectable or metastatic STS^18^. Anlotinib is the first drug to receive approval for the treatment of advanced STS in China^19^. Nevertheless, these patients have an OS less than 12 months, as well as cancer pain in local lesions, poor quality of life, and low physical strength scores^13^.

As the consequence of several minimally invasive disciplines, radioactive ^125^I seed implantation can deposit ^125^I particles into the tumor through puncture needle under the guidance of imaging. In this procedure, the particles play a radioactive role within the tumor and prolong the activity time, which can significantly decrease the side effects compared with other radiotherapy methods. Furthermore, this is a more accurate and comprehensive treatment with radiotherapy as the background compared with other similar methods. The application scope includes a variety of malignant tumors, including lung cancer, pancreatic cancer, liver cancer, bone metastases, as well as a variety of metastatic lymph node and soft tissue tumors^5,20–26^. Huang et al.^25^ reported the application of ^125^I seed implantation in the treatment of metastatic and recurrent head and neck tumors, and the results showed that the effective rate was 83.3% at 4 months after the surgery, and the OS was 31 months. Mo et al.^5^ reported the effect of ^125^I seed implantation combined with second-line chemotherapy for the treatment of metastatic STS. Although the results showed that the OS was 16.9 ± 5.1 and 12.1 ± 4.8 months, respectively, there was no statistical difference in the OS between the two approaches. However, the experimental group showed a higher local control rate, as well as a higher symptom relief rate and a better quality of life score, which seem to be more important to patients with advanced STS. Lastly, Yao et al. reported the safety and effectiveness of ^125^I seed implantation in the treatment of recurrent or metastatic STS in children^26^.

This study obtained a good local control rate. The tumor control rate was 93.5%, and the effective rate was 61.3% at 6 months after the operation. The good results in this study were mainly due to several reasons. Firstly, because the half-life of radioactive ^125^I particles is 2 months, the radioactive particles have a longer lasting effect compared with external radiation, and this can induce tumor cell apoptosis through the biological effects that the radioactive particles produce. The effective distance range of ^125^I particles can also significantly reduce radiation-induced damage of surrounding tissues, which is a key characteristic of ^125^I particles. Secondly, 3D template-assisted seed implantation is more accurate^27–30^. It reduces intraoperative bleeding and relative movement, and it can not only shorten the operation time and the number of times needed to adjust the puncture needle during repeated operations, but also ensure the precise control of the target dose based on the control of the tumor target volume^30^. The establishment of a reasonable treatment plan before surgery and the accurate execution of the treatment plan during the operation can effectively control the distribution of radiation doses, especially the distribution of tumor marginal doses, which is equivalent to the safe margin of the surgical resection^9–11^ and is also the basis for tumor recurrence and metastasis.

The application of 3D printing technologies stipulates that no zone is forbidden in seed implantation, as it avoids blood vessels and organs during the operation, thereby making the ^125^I particle distribution within the tumor more uniform and the radiological dose more reasonable, as well as eliminating the occurrence of the cold area of the tumor local dose. The goal of this approach was equivalent to surgical resection, and the local tumor control rate was further improved. The results of this study also showed that the relevant dosimetry indicators verified after the operation were not statistically different from those analyzed before the operation, which further supports the contention that the method can improve the rationality of the actual dose distribution. Presently, there is no large sample study on the application of this technology in the treatment of advanced STS. At the same time, although our results showed the safety and effectiveness of this method in the treatment of advanced STS, it is unclear whether other treatments combined with CT-guided 3D template-assisted radioactive ^125^I seed implantation can further improve the OS of patients with STS. Therefore, the prospective stratified analysis of a large cohort is still needed to further confirm the effectiveness of this method.

## Data Availability

All data generated or analyzed during this study are included in this published article.

## Abbreviations

STS: Soft tissue sarcoma
OS: Overall survival
BTPS: Brachytherapy treatment planning system
PTD: Planned target dose
PD: Prescription dose
PTV: Planned target volume
CTV: Clinical target volume
CI: Conformal index
EI: External index
KPS: Karnofsky physical score
CR: Complete remossion
PR: Partial remission
SD: Stable disease
ORR: Objective remission rate
DCR: Disease control rate
